# Book and Pamphlet Notices

**Published:** 1860-07

**Authors:** 


### Editorial.

### BOOK AND PAMPHLET NOTICES.

THE DISEASES OF THE EAR: THEIR NATURE, DIAGNOSIS, AND TREATMENT. BY JOSEPH TOYNBEE, F. R. S., Fellow of the Royal College of Surgeons of England; Aural Surgeon to St. Mary's Hospital; Aural Surgeon to the Asylum for Idiots; Consulting Aural Surgeon to the Asylum for the Deaf and Dumb, etc.; London. Philadelphia: Blanchard & Lea. From S. C. Griggs & Co., 39 and 41 Lake street, Chicago.

The literature of aural surgery is less extensive than that of almost any other branch of medical science. Comparatively few eminent men in our profession have devoted their best energies to the investigations of the diseases of the ear and their therapeutics; consequently few satisfactory and reliable works have ever been written on these subjects, and of these few, with one or two exceptions, those of recent date, alone, are of especial value. It must be admitted that these last are, in many respects, imperfect, and that any valuable addition to their number should be favorably received by the profession.

The work of Mr. Toynbee is undoubtedly, upon the whole, the most valuable production of the kind in any language. The author has long been known by his numerous monographs upon subjects connected with diseases of the ear, and is now regarded as the highest authority on most points in his department of science. Probably no one in England, or on the continent of Europe, has accomplished so much in the pathological anatomy of the ear, as Mr. Toynbee. Mr. Wilde, of Dublin, so well known as the author of one of our latest and best works on Diseases of the Ear, says of him, that "his labors and investigations have effected more for aural pathology than those of all his predecessors, either in England or on the continent. Accustomed to habits of minute dissection, patient research, and careful observation, he has accumulated a mass of facts upon the morbid anatomy of the organs of hearing, that must lay the foundation for a more rational mode of treating the diseases of those parts, than has hitherto been resorted to." It is to be hoped "he will long continue to prosecute, with the same avidity, the same honesty of purpose, and an equal amount of critical acumen, his valuable researches."

After twenty years of patient labor, devoted to careful study, to an extensive hospital and private practice, and to the minute dissection of *two thousand* ears, he has produced a work which cannot fail to add to his reputation and extend the knowledge of diseases of the ear in the profession.

The first chapter is devoted principally to a consideration of the "Mode of Investigating the Diseases of the Ear," and the "Method of Dissecting the Ear."

The second chapter contains observations upon the anatomy, uses, and diseases, of the external ear, (auricle,) and although not without interest is perhaps of less practical importance than other portions of the work.

The six following chapters are devoted to the anatomy, pathology, and treatment of diseases of the external meatus. Inasmuch as these affections are quite common, and often lead to unfortunate consequences, their diagnosis and treatment should be carefully studied by every physician. Yet too many of our profession betray great ignorance, or, rather, carelessness, in their management of such diseases. Instances are not unfrequent where physicians have treated diseases of the external meatus without even an examination. We have known several cases, where, without an examination of the ear, attempts have been made, by blistering and leeching, to relieve pain and deafness caused by the presence of impacted cerumen. Everything of this kind is wrong, and is certain to bring discredit, not only upon the surgeon individually, but upon the profession at large. It as an excellent rule, applicable in all cases of aural as well as of general practice, never to treat a patient without a thorough examination of the organ affected.

Facility in conducting an accurate examination of the ear, and in recognizing its abnormal conditions, is only acquired by long-continued practice. It is of the utmost importance to have a clear idea of the normal appearance of the external meatus, and to be able to distinguish individual peculiarities, *resembling* the effects of disease, from abnormal conditions. This can only be accomplished by the careful inspection of the ears of a large number of healthy individuals.

A good view of the lining membrane of the external meatus, and of the membrana tympani, can often be obtained by simply pulling the upper portion of the auricle upwards and backwards, and allowing the rays of the sun to fall directly into the ear. Usually, however, the assistance of the speculum is required. The instrument adopted by Mr. Toynbee is a modification of the ordinary conical tube introduced by Gruber and Wilde. The portion which enters the meatus is of the same circumference throughout, but oval, like a flattened cylinder, corresponding to the oval shape of the outer meatus. "In order to hold the speculum more firmly, it is desirable that the expanded portion should be somewhat flattened; and this flattening should be at right angles with that of the small extremity."

The "forceps speculum" is much less convenient than the instrument just described, especially in cases of operation, and is now little used. When we consider the structure of the osseous portion of the meatus, it will be at once apparent that nothing can be accomplished by attempting to dilate it, by means of the "valvular speculum."

The practitioner will find great convenience, when the sun is not in a favorable position for an examination in having a mirror so adjusted outside his office window as to reflect the sun's rays horizontally into the ear of a patient, sitting in any part of the room. Although the direct rays of the sun are always preferable, it is sometimes necessary, when the sun is obscured, to employ artificial light. A good view of the cavity of the external meatus can be obtained by throwing the light of a good lamp or gas burner through the speculum into the ear by means of a concave reflector, having a small opening in the center, through which the surgeon can look. Specula with magnifying lenses are sometimes employed with advantage.

It should be remembered, in every case where there is a discharge from the meatus, that the ear should be gently syringed, and carefully dried by means of a small tuft of cotton, before a satisfactory examination can be made. With these precautions the surgeon can detect the presence of any disease peculiar to the meatus.

In treating of the diseases of the external meatus, the author furnishes the student with important practical information on the following subjects; foreign bodies and accumulations of cerumen in the meatus and their removal; acute and chronic inflammation with or without discharge and caries of bone; polypi; osseous and molluscous tumors.

The subject of the next three chapters is the Membrana tympani—its structure and functions; the inflammations of its layers; perforation from ulceration and injury; and the use of artificial membrana tympani.

A tabular view is given of the abnormal condition of the membrane in dissections of 1013 diseased ears. The membrana tympani is an important portion of the organ. Its diseases, analogous, in many respects, to those of the cornea, are somewhat grave as regards the sense of hearing. Extensive perforation is almost uniformly attended, to a greater or less extent, with obstinate discharge and difficulty of hearing. These affections, therefore, are of great importance, and should receive the careful attention of all who attempt to treat them.

A single chapter—one, however, of great interest—is devoted to the physiology and pathology of the eustachian tube. In examining the ear with reference to the diseases of this canal, several facts should be born in mind; that the faucial extremity of the tube is closed except during the act of deglutition; that deglution opens this orifice by the contraction of the tensor and levator muscles of the palate, which take their origin from the soft tissues around it; that either deglutition or a forced expiration with the mouth and nostrils closed, sends a small quantity of air through the tube into the cavity of the tympanum, producing a slight projection of the membrane tympani outward; this is normally attended by a faint crackling sound, which can be distinctly heard by the surgeon, by placing in his ear one end of a flexible tube, (otoscope,) the other end being inserted in the ear of the patient.

The author describes, with great clearness, the symptoms and appearances by which we may be able, in many instances, to decide whether obstructions of the tube exist at its faucial or at its tympanic extremity. With a careful examination of these symptoms and appearances, he is disposed to consider the *Eustachian catheter as entirely useless* in diagnosis, but sometimes advantageous in treatment.

It appears in a table found in this chapter, that in 1523 dissections, only 29 cases were found with diseased condition of the tube.

"The cavity of the Tympanum" is the subject of the next two chapters, which are also sub-divided into sections, upon: The Anatomy and Pathology of the parts within the Cavity; Diseases of its mucous membranes, with or without ulceration and affections of the Portio Dura Nerve, bone and brain; Anchylosis of the ossicles; Formation of membranous bands of adhesion. To illustrate the history of these various forms of disease and their treatment, several interesting cases are reported, which are followed by a discription of numerous experiments showing the physiological action of the ossicles in conveying sonorous undulations to the auditory nerve. A remarkable and valuable table is also given, exhibiting the pathological changes, which were found in 1015 dissections of this portion of the ear; among which are 151 cases with abnormal products in the cavity; 365 with morbid affections of the mucous membrane; 202 with false membranous bands uniting different portions of the Tympanum; 30 with abnormal condition of the malleus, 189 of the stapes and 31 of the incus. Accurate diagnosis of these affections during life, as also their successful treatment, must of course very often be found impossible. And yet the author shows that in some of these diseases, however obscure they may at first appear, the experienced surgeon may learn enough from the symptoms and history of the case to form a correct diagnosis.

A single chapter is devoted to the diseases of the Mastoid cells. It contains much valuable knowledge regarding these diseases, particularly those of an inflammatory nature, which are liable to terminate in ulceration of the mucous membrane, necrosis of the bone and affections of the coverings and substance of the brain. The author expresses his opinion regarding discharges from the ear in reference to life insurance. He says: "No doubt a discharge from the ear should always be regarded with suspicion; an opinion which is borne out by an inspection of the following table (which is given) showing the relation between the duration of the discharge and the acute symptoms," which very often terminate fatally. We would call especial attention to the importance of healing diseases with discharges from the ear as speedily as possible. This is contrary to the popular idea and to the opinion of too many physicians and surgeons. Careful study of the works of Mr. Toynbee and Mr. Wilde will convince any one that a continuation of the discharge is sometimes not without danger to life. "*So long as otorrhoea is present, we can never tell how, when or where it will end, or what it may lead to.*" It is certainly loathsome to the patient and his friends and destructive to the sense of hearing.

The 15th and 16th chapters are devoted to "The Diseases of the Nervous Apparatus" and to "Nervous Deafness." The chapters are filled almost entirely with the history of cases, although in the majority of instances, diseases of this kind, like amaurosis is not benefited by treatment, yet great care should always be exercised in the examination of a patient, since nervous deafness is not unfrequently produced by indigestion, mental and physical fatigue and excitement and by dibility, fever and hemorrhage. Suitable treatment adapted to these several conditions of the system will semetimes relieve the local disarrangement of the auditory nerve.

The remaining chapters on "Malignant Diseases of the

Ear," "The Deaf and Dumb" and "Ear Trumpets and their uses," are interesting and worthy of a careful perusal. An appendix is added containing a "list of published papers on the structure, functions and diseases of the Ear," with references to the journals in which they can be found.

Mr. Toynbee's work as we have already said is undoubtedly the most reliable guide for the study of the disease of the ear in any language, and should be in the library of every physician.

"The volume will be found thoroughly illustrated with a large number of original wood engravings, elucidating the pathology of the organs of hearing, instruments, operations, &c., and in every respect, will be one of the handsomest specimens of mechanical execution issued from the American press."

We must say, however, that some of the engravings are very imperfect and almost entirely fail to assist the reader in forming an adequate idea of the specimen illustrated. Any one who has studied the works of Dalrymple, Jaeger and Sichel on Diseases of the Eye, and examined the numerous anatomical and pathological plates, so remarkable for beauty of drawing and delicacy of coloring, cannot but regret that Mr. Toynbee had not endeavored to furnish colored illustrations of a high artistic character, exhibiting the appearance of every part of the ear in health and disease. They would have been an invaluable addition to his work. E. L. H.

☞ We have received the following announcement of the advent of a new medical book:

THE MEDICAL USES OF ELECTRICITY IN THE TTEATMENT OF NERVOUS AFFECTIONS.—A new and important medical work, issued by Messrs. Ticknor & Fields. It is a thoroughly systematic work, of over 700 pages, and finely illustrated with over 100 cuts, showing not only the best methods for the therapeutical employment of electricity in the various nervous diseases, but also showing the anatomy of the parts, (nerve-trunks and muscular fibres,) liable to be involved; moreover presenting a concise view and means of diagnosis of the great variety of nervous affections met with in every-day practice. This work is from the pen of Alfred C. Garratt, M. D., of Boston, who, of late years, has made this difficult department of medicine his speciality. It is addressed to medical students, and is dedicated to Dr. John Homans, President of the Massachusetts Medical Society.

